# Dual intracranial infection with *Nocardia farcinica* and *Cryptococcus neoformans* diagnosed by next-generation sequencing in a patient with nephrotic syndrome: A case report

**DOI:** 10.1097/MD.0000000000030325

**Published:** 2022-09-02

**Authors:** Hongmei Ma, Xiangbo Wang, Heli Yan, Qing Liu, Dan Yang, Tingting Bian

**Affiliations:** a Department of Neurology, Beijing Fengtai You’anmen Hospital, BeijingChina; b Department of Neurology, Xuanwu Hospital of Capital Medical University, Beijing, China.

**Keywords:** *Cryptococcus neoformans*, intracranial infection, next-generation sequencing, *Nocardia farcinica*

## Abstract

Intracranial infections are associated with high morbidity and mortality in immunocompromised patients, due to delayed diagnosis and treatment. Establishing a rapid, accurate diagnosis and a precise therapeutic regimen is crucial for management of the patients. Our report described a rare intracranial infection of patient with nephrotic syndrome.

**Patient concerns:**

A 66-year-old woman with a history of nephrotic syndrome presented symptoms in central nervous system for 1 month, followed by headache and fever over several days.

**Diagnosis:**

Neurological examination, brain imaging, and cerebrospinal fluid (CSF) tests exhibited resemblance to intracranial infection. Subsequently, CSF cultures confirmed the presence of *Cryptococcus*. Fortunately, next-generation sequencing revealed the concomitant infection with *Nocardia farcinica* in addition to *Cryptococcus neoformans*.

**Interventions:**

The treatment with intravenous fluconazole combined with amphotericin could not immediately ameliorate her symptoms. The patient’s condition improved significantly with minimal deficits after timely administration of antibiotics against *N farcinica*.

**Outcomes:**

One month later, cranial MRI indicated that basal ganglia lesions ameliorated. The patient has recovered well.

**Lessons subsections:**

To our best knowledge, this is the first case report of intracranial infection caused by both *N farcinica* and *C neoformans* in a patient with nephrotic syndrome. Remarkably, extensive application of next-generation sequencing can facilitate investigation on the potential role of various pathogenic organisms in infectious diseases.

## 1. Introduction

Nervous system can be infected by various pathogens including bacteria, fungi, and viruses, causing potential life-threatening diseases. Central nervous system (CNS) diseases, such as meningoencephalitis, cephalitis, and brain abscesses, contribute to high death rates of the patients.^[1,2]^ Early identification of the causative agents is vital for the treatment of CNS infections despite the great difficulties. Around 30% to 40% of the CNS infections (e.g., meningitis and encephalitis) can be identified through cerebrospinal fluid (CSF) culture.^[3]^ Nevertheless, the diagnostic rate of CSF culture for meningitis is only approximately 5.4% to 24.3% in developing countries according to the latest reporters.^[4,5]^ Moreover, many hospitals are unable to culture CSF, which further decreases the positive detective rate. Other methods for CNS infection diagnosis such as Xpert MTB/RIF Ultra, loop-mediated isothermal amplification in addition to tissue biopsy promote the detective efficiency to some extent, while exhibiting several limitations.^[6,7]^ Recently, great progress has been made in metagenomics next-generation sequencing (NGS), which can be applied to precise medicine. Metagenomics NGS is semiquantitative that can quickly identify a variety of pathogens,^[8–14]^ serving as a reliable approach for potential pathogenic microorganisms detection that are involved in neural infectious disorders.^[2–4]^

*Cryptococcus neoformans*, formerly called *Saccharomyces neoformans*, was first isolated from peach juice by Sanfelice in 1894,^[5]^ and the first case of human cryptococcosis was reported in the same year.^[6]^ *Cryptococcus neoformans* is the predominant fungus that leads to meningoencephalitis in CNS globally.^[7]^ It was recently estimated that approximately 1 million new cases of cryptococcal meningitis are annually diagnosed, which resulted in a mortality rate of higher than 60% within 3 months after the first infection.^[8,9]^

We herein present a case of meningoencephalitis where Gram staining and culture of pathogenic microorganisms were nondiagnostic or discrepant, while NGS-based DNA sequencing suggested co-infection by *N farcinica* and *C neoformans*.

## 2. Case presentation

A 66-year-old woman with a history of prolonged cognitive decline, unstably walked for 1 month and presented headache, fever, and somnolence that lasted for 3 days. The patient’s medical history revealed that she has been suffering from nephrotic syndrome since March 2018 and long-term treated with prednisone and immune-suppressants including 25 mg (bid) cyclosporine as well as regularly intravenous cyclophosphamide. The patient had no history of diabetes mellitus, tuberculosis, hepatitis, drug allergy, or major trauma and had not traveled recently. Three months prior to admission, she experienced headache with fever and was diagnosed with hemorrhagic fever with renal syndrome, complicated by viral myocardial damage and fungal infection, at a local hospital, and received a 3-week antiviral (ribavirin at 0.5 g intravenously [i.v.]) and 6-day antifungal (fluconazole at 0.2 g i.v.) treatments. Following brief improvement, her diseases developed into progressive sluggishness, memory deterioration, and unsteady gait, which lasted for 1 month. Three days earlier, the patient experienced recurrent headache with fever, so she was admitted to our hospital for further investigation.

**Figure 1.**
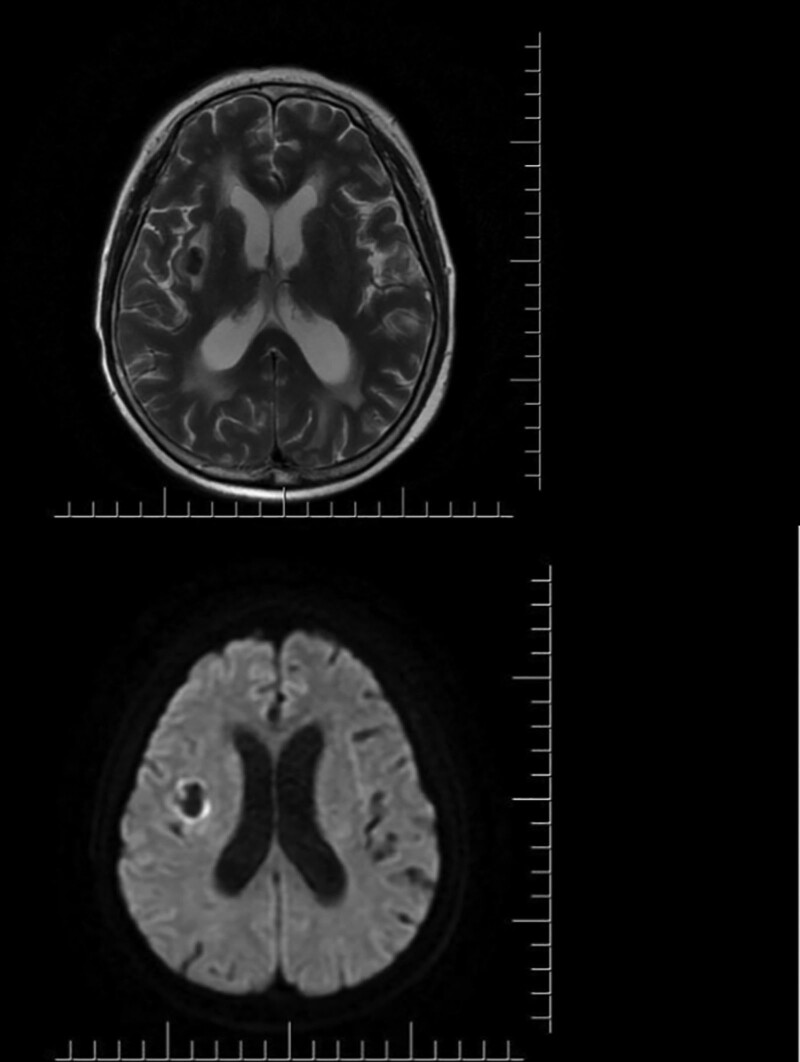
MRI: T2: low signal in the right outer sac, DWI: annular hypersignal in the right outer sac. DWI = diffusion-weighted imaging, MRI = magnetic resonance imaging.

The patient was febrile (38.0°C) on admission, with a heart rate of 84 beats per minute, blood pressure of 163/103 mm Hg, and respiratory rate of 20 breaths per minute. Neurological examination suggested higher cortical function degeneration and speech disorders. Neck stiffness was found, while no reflex of Brudzinski or Kernig sign was observed. Moreover, Babinski pathological reflection of the right part of her body was positive. The lower left extremity showed 5- strength and other limbs exhibited 4 strength, while her muscular tension displayed normal. Ataxia assessment was not performed because the patient could not cooperate. Brain magnetic resonance imaging (MRI) examination indicated multiple lacunar infarction, mucosal thickening in bilateral ethmoid sinus, and hydrocephalus accompanied by interstitial edema (Fig. 1).

Laboratory examinations showed that she suffered from moderate anemia (hemoglobin 91 g/L) and electrolyte disturbances (potassium, 3.49 mmol/L; chlorine, 116 mmol/L; sodium, 151.8 mmol/L; cholesterol, 5.89 mmol/L), as well as enzymatic disturbances (gamma-glutamyl transferase, 66 U/L; β_2_-macroglobulin, 3.64 mmol/L; creatinine [enzymatic], 106 µmol/L; total protein, 45 g/L; albumin [bromocresol green], 26.8 g/L; lactic dehydrogenase, 363 U/L). Routine urine testing also revealed positive urine occult blood, urine protein, and erythrocyte, suggesting the diagnosis of renal insufficiency.

The routine CSF tests performed on admission showed clear appearance, opening pressure of 250 mm H_2_O, whole blood cell (WBC) of 80 cells/ mm^3^, protein of 0.545 g/L, glucose of 2.23 mmol/L, and chloride of 119.1 mmol/L. Gram and acid-fast staining were negative. Notably, CSF culture identified *Cryptococcus* species sensitive to fluconazole, therefore, antifungal treatment with intravenous fluconazole (i.v. 600 mg/d) combined with amphotericin (i.v. gradually increasing dosage from 2 to 5 mg considering renal function impairments) was provided.

Three days later, the patient was still febrile, experiencing distending sensation and headache. The CSF specimen was processed for next-generation sequencing (NGS) analysis, which yielded 6844 reads mapping to *C neoformans* strain (GenBank accession no. JF797311 and GQ85380) and 310 reads matching with *N farcinica* strain (GenBank accession no. JF797311 and GQ85380), while only 8 reads were from human gammaherpesvirus-4. Based on these results, empirical antibiotic regimen consisting of sulfamethoxazole and ceftriaxone targeting *N farcinica* was administrated, and meanwhile, the dosage of amphotericin was changed to 10 mg.

Fourteen days after admission, the patient’s ability to speak and her muscle strength were significantly improved. Lumbar puncture was performed to reanalyze the pathogens and biochemical parameters in the CFS specimen of the patient, which detected total cell count of 56 cells/ mm^3^, WBC of 12 cells/ mm^3^, protein of 64 mg/dL, glucose of 2.82 mmol/L, and chloride of 116.5 mmol/L. Although no microorganism was positive for Gram staining, *C neoformans* was detected by NGS analysis. Furthermore, the blood and CSF cultures were positive for capsular polysaccharide antigen of *Cryptococcus* (with a titer > 100 μg/L). Repeated MRI displayed abnormal signals in basal ganglia region and lobus insularis on the right side of the brain, implying a high possibility of meningoencephalitis caused by infection. Therefore, the current therapeutic regimen was maintained.

On the 37th day, remarkable improvements were observed in the clinical manifestations and corresponding CSF analytical outcomes, including decreased WBC count to 14 cells/ mm^3^, reduced protein level to 38 mg/dL, and negative Gram staining and culture results. MRI examination suggested that there were reduced basal ganglia lesions in right insula. Therefore, intravenous antibiotic treatment targeting *N farcinica* was discontinued and modified to oral minocycline treatment (100 mg bid), which was administrated for at least 1 year to decrease the risk of relapse. In particular, minocycline was used because of sulfonamide intolerance of the patients and informed consent for side effects was obtained from the patient and her relatives. Meanwhile, culture results were still positive for capsular polysaccharide antigen of *Cryptococcus sp.* (with titers > 100 μg/L in blood and > 73.25 μg/L in CSF), and 2 *Cryptococcus* spores were identified in the CSF culture by India ink staining. Forty-six days after admission, the patient was discharged while remaining for amphotericin (i.v. 20 mg/d) and fluconazole (i.v. 400 mg/d) treatment for 2 to 3 weeks. antifungal therapy was suggested to be replaced by oral antibiotic treatment following 3 consecutive negative results from CSF India ink staining.

One month after discharge, the patient was subjected to cranial MRI, which indicated that her basal ganglia lesions were ameliorated. The patient has recovered well.

## 3. Discussion

*Cryptococcus neoformans* infections primarily occurred in immunocompromised individuals such as those with chronic lung, renal, or hepatic diseases, as well as in patients receiving immunosuppressive regimens following organ transplantation.^[10]^ In this case, the patient had a nephrotic syndrome and was chronically administrated with immunosuppressants including cyclosporine and cyclophosphamide, which increased the risk of *C neoformans* infection.

The etiology of human diseases is usually characterized by coexistence of diversified pathogens. Unfortunately, conventional culture-based or molecular approaches cannot effectively identify such cooperative microbiological compositions in some cases.^[11]^ NGS offers a relatively unbiased approach to profile complex bacterial communities in individual specimen and detect the microbial components even with very low abundances,^[12,13]^ which has been increasingly commercialized and accessible to clinical laboratories. During the early admission of this patient, neurological examination, brain imaging, and CSF tests all exhibited resemblance to intracranial infection, and CSF cultures confirmed the presence of *Cryptococcus*. However, treatment with intravenous fluconazole combined with amphotericin did not immediately ameliorate her symptoms. Fortunately, NGS analysis was then performed to comprehensively detect the infectious pathogens by capturing microbial genomes amplified from human CSF specimen. In accordance with the results from *Cryptococcus* antigen test, a majority of sequencing reads were aligned with *C neoformans* genome. In addition, *N farcinica* was also identified with high abundance while only a small portion of the sequence reads were aligned with human gammaherpesvirus-4 genome. Zhou et al^[15]^ have recently documented a idiopathic thrombocytopenic purpura with brain abscess using the similar technique metagenomics NGS to detect CSF, and the *Nocardia* infection was also found. Accordingly, NGS of CSF samples might be a promising method conducive to the diagnosis of infectious diseases.

Up to now, a total of 54 *Nocardia* species have been identified as clinically significant microorganisms,^[14,16]^ among which *N farcinica* is one of the most common pathogens causing nocardiosis.^[17]^ Immunosuppressed and elderly populations are more susceptible to nocardiosis,^[16,18]^ and the prognosis of nocardiosis is usually poor in patients suffering from several other underlying diseases such as stroke. Nocardiosis can occur in a wide array of organs (i.e., brain, lung, skin, and eyes). Following empirical administration of antibiotic regimen consisting of sulfamethoxazole and ceftriaxone targeting *N farcinica*, the symptoms of the patient were significantly ameliorated.

Given the molecular results, we speculated that the pathogens could be eliminated by the antifungal therapy prior to specimen collection, and thus *C neoformans* might be undetectable through Gram staining or culture-based approach. Alternatively, it is also possible that the pathogens were at extremely low abundances, which could only be detected via polymerase chain reaction amplification but not the conventional culture-based method. In summary, by reporting this case, we highlight the importance of extensive employment of NGS analysis in accurate diagnosis of infectious diseases caused by multiple pathogens. Meanwhile, NGS may also be applied to assess the overall composition of a polymicrobial community in the specimen of patients.

## Author contributions

Hongmei Ma put forward the ideas of this article, wrote this article, analyzed the data, and reviewed the manuscript; Xiangbo Wang and Heli Yan reviewed the literature and collected the data; Qing Liu, Dan Yang, and Tingting Bian helped for analysis and interpretation of data; all authors issued final approval for the version to be submitted.
